# Development and evaluation of an online integrative histology module: simple design, low-cost, and improves pathology self-efficacy

**DOI:** 10.1080/10872981.2021.2011692

**Published:** 2021-12-14

**Authors:** Daniel T. Schoenherr, Mary O. Dereski, Kurt D. Bernacki, Said Khayyata, Stefanie M. Attardi

**Affiliations:** a Department of Foundational Medical Studies, Oakland University William Beaumont School of Medicine, Rochester, MI, USA; b Family Medicine Residency, National Capital Consortium, Fort Belvoir, VA, USA; c Department of Anatomic Pathology, Beaumont Health, Royal Oak, MI, USA; d Department of Pathology, Oakland University William Beaumont School of Medicine, Rochester, MI, USA

**Keywords:** Medical education, integration, histology, pathology, online module

## Abstract

Integration of core concepts is an important aspect of medical curriculum enhancement. Challenges to improving integration include the risk of curtailing the basic sciences in the process and the push to decrease contact hours in medical curricula. Self-paced learning tools can be developed that deliberately relate basic and clinical sciences to aid students in making interdisciplinary connections. The purpose of this project was to develop, implement, and evaluate a self-paced learning module that would be applicable to integration of different disciplines in medical education. The module was intended to improve integration between histology and anatomic pathology before a respiratory pathology laboratory session. Qualtrics XM, a survey software commonly available at educational institutions, was used in a novel manner to create the module. Module activities included pre- and post-module quizzes; four short videos emphasizing normal histological features and recalling associated pathologies; three categorization activities designed for students to recognize normal versus abnormal characteristics of lung specimens; and post-activity feedback. Preliminary data from first-year medical students showed that post-module quiz scores were significantly higher than pre-module quiz scores (*p *< 0.001) and that module users’ pre-laboratory pathology self-efficacy was significantly higher than non-users (*p *< 0.05). These data suggest that module use facilitated short-term knowledge gain and improved pathology self-efficacy before the laboratory session. Online modules can be developed affordably using Qualtrics XM to integrate anatomical sciences with other disciplines, while providing students interactive learning resources without increasing contact hours. The module presented in this report focused on normal versus abnormal morphology, guiding students through recognizing the continuum from healthy to disease states before learning about the pathologies more in depth. A similar module design would likely be effective in integrating other disciplines in medicine, especially in disciplines that require recognition of changes in morphology.

## Introduction

### Conceptual framework for integration of basic sciences in undergraduate medical education

Integration involves teaching separate disciplines in combination with each other in a way that relates to previous learning and is relevant to future application for the learner [1]. The importance of integrating sciences has gained new attention in medical education and practices, especially in the rising era of personalized medicine and prevention where it has become necessary to combine understanding of multiple fields of study [2,3]. In 2010, the Carnegie Foundation called for changes in medical education, including promotion of integration, to meet the diverse needs of healthcare [4]. Liaison Committee on Medical Education-accredited institutions are responsible for the design, management, integration, evaluation, and enhancement of the medical curriculum with processes that include horizontal and vertical integration [5]. Horizontal integration is the integration of disciplines taught within themselves or within each year of the curriculum (e.g., the integration of histology and pathology within an organ system course). In comparison, vertical integration is the incorporation of disciplines taught in different phases, such as combining clinical-based learning with basic sciences [6,7].

Acquiring foundational knowledge is necessary for medical students before learning more advanced topics and clinical applications. Students, especially adult learners, are more willing to put effort into learning when they understand its relevance [8]. By integrating basic science material with clinical problems, learners are more engaged in the topics [7,9]. Students able to conceptualize the material they have learned, rather than memorizing it, have seen more success in integrating basic sciences and clinical concepts in their diagnostic reasoning [6,7,10–12]. Challenges to improving integration include the risk of diminishing the importance of the basic sciences in the process [7] and the push to decrease contact hours in medical curricula [13]. In addressing these challenges, learning tools can be developed that deliberately relate curricula to aid students in making connections between basic sciences and clinical applications or between normal morphology and disease states [14]. Such learning tools can be self-paced and made available online for students’ independent study time to avoid increases in contact hours [13]. The purpose of the current project was to develop, implement, and evaluate a concise, self-paced learning tool, using widely familiar survey software, to improve integration between histology and anatomic pathology at Oakland University William Beaumont School of Medicine (OUWB). The approach was piloted using a respiratory system course and was intended to be applicable to integration of other disciplines in medical education, such as anatomy and radiology or embryology and developmental anomalies.

### OUWB curriculum

OUWB is an allopathic medical school located in the American Midwest. The four-year medical curriculum (M1-M4) has approximately 125 students in each class. The anatomical sciences are introduced in the preclinical years (M1-M2) prior to clinical rotations (M3-M4). A foundational course in the anatomical sciences (54 h lecture; 78 h laboratory) is offered in the first semester of the M1 year, encompassing clinically-oriented gross anatomy, cadaveric dissection, basic tissue histology, early embryology, and radiology. Six hours of the laboratory experience in this course are designated for histology, during which students work in small teams to follow an instructor-written document to guide their identification of histologic structures using virtual microscopy (VM). The remainder of the M1 year and the M2 year are organized into integrated, organ system-based courses during which students learn the basic and clinical sciences for a given body system. Histology education continues into the organ system courses (23 h lecture, 25 h VM laboratory), expanding from the basic tissue types to include detailed topics on the particular organ system. The learning of histologic concepts and normal microscopic images serves to form a foundation for pathology instruction in the organ system courses (69 h lecture; 26 h laboratory). Pathology laboratories are organized into clinically-themed stations facilitated by a pathologist or pathology resident. Students rotate in groups through the stations, discuss clinical cases with their instructors, and identify pathologies. The types of specimens available for study vary between the system courses and may include virtual and light microscope slides, gross pathologic specimens, and images.

The Respiratory course spans five weeks at the conclusion of the M1 year, covering normal and abnormal functions of the respiratory system. Class sessions for the study population included a respiratory histology lecture (1 hour) and laboratory session (1.5 hours) during the first week of the course. There were six pathology lectures (6 hours) delivered over the second through fourth weeks and an ungraded respiratory pathology laboratory session (1.5 hours) in the fourth week. The laboratory consisted of ten cases divided into four stations, each 20 minutes in length. Two faculty pathologists and two pathology residents served as facilitators for the four stations, and each station included 12–16 students. Case topics included pulmonary hamartoma, carcinoid tumor, small cell carcinoma, adenocarcinoma, sarcoidosis, granulomatosis with polyangiitis, *Coccidioides immitis* infection, *Pneumocystis jirovecii* infection, Cytomegalovirus infection, and tonsillar squamous cell carcinoma. Clinical histories and laboratory exercise questions were provided for each case on a paper worksheet. Students were asked to examine the virtual slide(s) for each case and determine whether the pathologic process was neoplastic or non-neoplastic. In developing a differential diagnosis, subsequent questions directed students to consider pertinent clinical details that would help refine the differential, such as symptoms, past medical history of immunosuppression, smoking history, or travel history. Students were then asked to determine what types of additional histologic stains (microbiologic or immunohistochemical) would help refine the differential diagnosis. In some cases of lung tumors, after the correct diagnosis was reached, questions relating to subsequent molecular testing or therapy were posed. Pathologist facilitators circulated amongst the students to help guide with location of diagnostic foci on the virtual slides and to assist with progression through the exercise questions.

### Problem

Based on observations by OUWB pathology faculty, there was a need in the Respiratory course to reinforce normal histology before the pathology laboratory session due to a three-week time gap between the two laboratory sessions. The histology and pathology instructors were aware of the topics covered in the other subjects, but there was limited explicit integration of the subjects, reflecting the step of ‘awareness’ on Harden’s integration ladder [14]. Nivala et al. [15] demonstrated that training students to understand the underlying model of normal compared to diseased tissue has been shown to improve recall, application, and interpretation of clinical details. They also demonstrated that students’ performance on diagnostic pathology examinations was predicted by the students’ prior knowledge of histology. Therefore, an increase in explicit integration between histology and pathology in the OUWB Respiratory course could improve students’ pathology laboratory experience.

Individuals born 1981–1999, reflecting the age range of OUWB students for whom this module was made, have been shown to prefer self-paced, interactive learning environments with immediate feedback on their learning progress delivered through an online environment [13,16–18]. Teaching strategies that use multiple modalities, have logical progression of material, signal emphasis of key concepts, and give learners more control while interacting with dynamic visualizations have been shown to help decrease cognitive load and improve learning of the material [19,20]. Increasing student engagement with hands-on, active learning activities, such as self-guided tutorials, can improve academic performance when compared to passive lecture instruction [2,19]. Therefore, the most productive and effective approach to integrating histology and pathology was determined to be an online self-guided histology module that contained multiple types of teaching processes, highlighted key concepts, and provided practice assessments with immediate feedback.

Digital technologies, including online modules, virtual microscopy, and other computer-based learning resources, have long been used to support learning histology and pathology in health sciences education. These resources have been shown to be more efficient [21,22], the preferred learning method for students [23], and equally [24–26] or more effective [23,27] for improving student performance compared to traditional class sessions. The use of computer-based modules for independent study has shown improved student performance and positive student feedback in integrated basic medical science curricula and in pathology instruction [2,19,28,29].

An online learning module is an interactive, self-guided learning tool that often contains content such as reading assignments, instructional videos, interactive tasks, and formative assessments to aid the students in learning course material [19,27]. The different types of instructional methods incorporated into a module provide new ways to practice material learned previously, explain complex concepts, provide links between topics, and present real-world clinical problems [27]. Modules can be developed at a low cost, making their use practical for all sizes of institutions. Upper level medical students can assist in module design, enabling opportunities for reinforcement for those students and vertical integration across the curriculum [2]. Modules may also be used for multiple years, creating consistency in instruction despite changes in faculty or teaching methods. Students may access the modules at any time as well, so they can be used as additional study resources at later times. Communication between instructors to adapt and incorporate each other’s content into their modules will help elevate a curriculum to the steps of ‘harmonization’ and ‘nesting’ in Harden’s integration ladder [14]. In demonstrating the success of using low-cost self-directed learning modules to aid in integration of histology and pathology while maintaining the current amount of contact hours, this method may be expanded to other courses and to other medical schools.

Developing new learning tools on module software can be very challenging for instructors with limited experience using instructional technology, and purchasing the licenses for such software can be expensive. This study describes the design of a module in Qualtrics XM, a widely-familiar software commonly available at institutions, demonstrating the ability to incorporate these online interactive learning experiences into medical education efficiently.

### Aims and objectives

The aim of this project was to demonstrate the use of Qualtrics XM survey software to create an online integrative learning module, and to determine if using this type of module affected medical students’ self-efficacy related to one of the integrated disciplines. Self-efficacy is one’s perceived abilities to meet the situational demands of a task [30]. The primary research objective was to determine how the use of a histology module of this nature affects students’ pathology self-efficacy in the context of a Respiratory System course. The secondary objective of this study was to ensure that students learned from the module. It was hypothesized that (1) module users will have significantly higher pathology self-efficacy than non-users; and (2) post-histology module quiz scores will be significantly higher than pre-module scores.

## Materials and methods

### Module development

To integrate histology and pathology in preparation for the pathology laboratory, the histology module reviewed concepts from the respiratory histology sessions with correlations to abnormal morphology learned in the pathology lectures. Pathological conditions often arise as a continuum of insults that result in a gradual change in structure and function from the norm. Training students to understand the underlying model of diseases improves recall, application, and interpretation of clinical details [15]. By focusing the module on normal versus abnormal morphology, it guided students through recognizing the continuum from healthy to disease states before learning about the pathologies more in depth. Qualtrics XM online survey software (Qualtrics, Provo, UT), a product that was not module-specific and was available at no additional cost due to a previously established university subscription, was used as the delivery platform. The software is used commonly by institutions for research data collection, is familiar to many educators and researchers, and is easy to learn. Its novel application in the current study demonstrated the expansion of its use for education.

**Figure 1.**
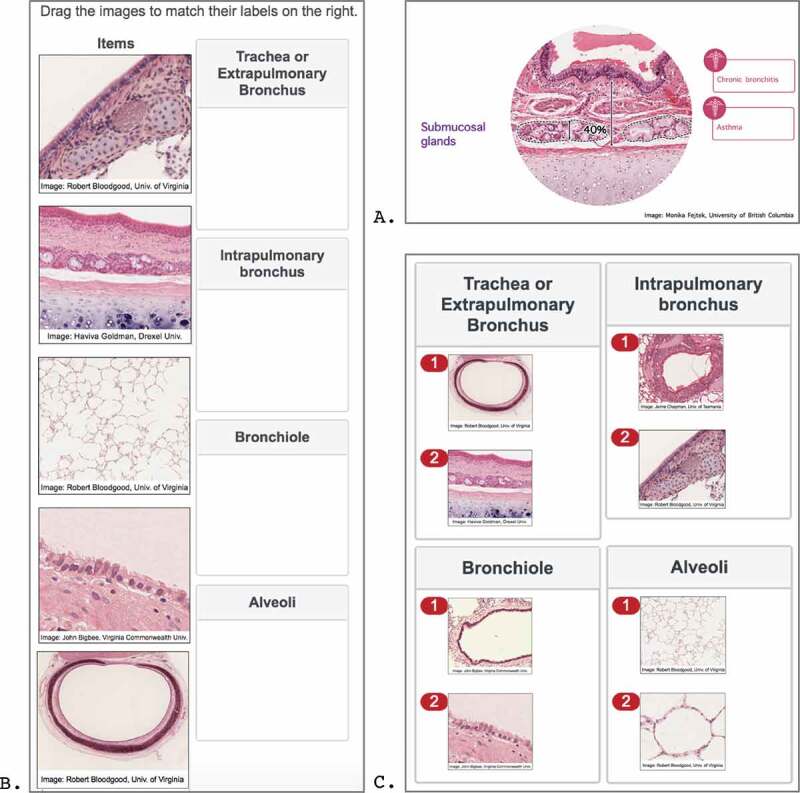
Module Screenshots. (A) Sample screenshot from the bronchus review video. In this frame, the normal thickness for the submucosal glands was reviewed and the names of two conditions were provided that involve changes to the thickness. (B) Sample screen shot from a drag and drop review activity. The user was instructed to move eight specimen images into the corresponding boxes. (C) Answers to drag and drop review activity shown in (B). Histology images were obtained from the Virtual Microscopy Database under an Attribution-NonCommercial-ShareAlike 4.0 Creative Commons License (CC). Image (A) was edited to add arrows and percent

The first section of the module displayed four short (1–1.5 minutes) videos on the topics of the respiratory epithelium, bronchi (Figure 1(A)), bronchioles, and alveoli. The videos were made by a histology faculty member (S.A.) in consultation with two faculty pathologists (K.B. and S.K.) to verify the pathology correlations, reflecting the steps of ‘harmonization’ (communication with other instructors to adapt outside content to their teaching) and ‘nesting’ (instructors incorporate content from other courses into their own teaching) on Harden’s integration ladder [14]. The purpose of the videos was to review these structures by emphasizing which histological features should and should not be present normally, and to recall specific pathologies associated with the named morphological features. Each narrated video began with a normal, low magnification image to orient the viewer to the structure of interest. Higher power views of specific areas of interest on the initial image were subsequently displayed and explained briefly. Text labels for the structures appeared in real time as they were discussed. Pathologies related to the structures were named and described briefly, with text labels appearing as they were discussed. The topics and content of each video are listed in Appendix 1. The visual materials for the videos were built in PowerPoint (Microsoft Corporation, Redmond, WA). Microscopy images were acquired from the Virtual Microscopy Database [31] and from Western University’s online Virtual Slidebox [32] with permission. The videos were recorded in Camtasia 3 for Mac (TechSmith, Okemos, MI) to create a voice-over-PowerPoint mp4 file. The lead investigator (D.S.), a medical student, tested the videos for audiovisual clarity and instructional pace. The videos were hosted on an unlisted YouTube channel (YouTube, San Bruno, CA) and embedded in the Qualtrics page for viewing; thus, the user could control the delivery pace and viewing time.

**Figure 2.**
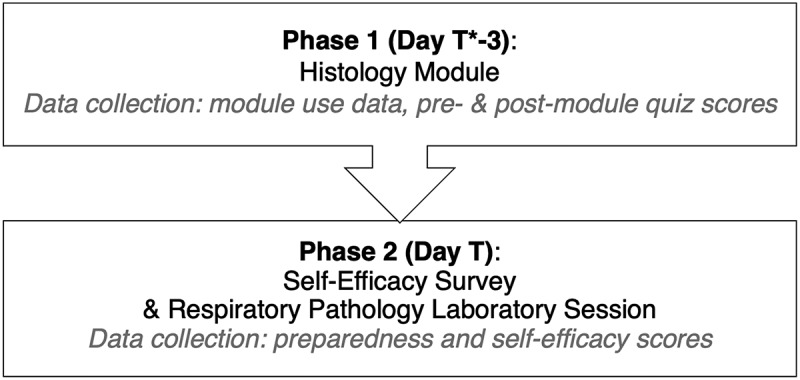
Study Timeline

The second part of the module consisted of three categorization activities designed for students to identify normal versus abnormal characteristics and patterns of lung specimen. In activities 1 (Figure 1(B)) and 2, the user was required to drag high and low magnification images of normal histological structures to match the correct text label and the correct functional description, respectively. Both of these activities were programmed using Qualtrics’ *pick, group, and rank* question type. In activity 3, the user had to indicate, using a radio button, whether the microscopic images provided were normal or pathologic. This activity was programmed using Qualtrics’ *matrix table* question type. The answers (Figure 1(C)) were displayed immediately following the activities to provide the user with immediate feedback.

### Implementation

First-year medical students were invited to use the module via an in-class announcement and by email. The online module was made available to students via a hyperlink sent by email and posted on the Respiratory course website three days prior to the pathology laboratory. The module did not replace any lecture time; it served as an optional, supplemental resource for histology review and pathology laboratory preparation. Thus, it did not increase contact hours or mandatory course work for the students.

### Evaluation

The protocol for the evaluation of this project was approved by Oakland University’s Institutional Review Board (IRB# 1237357). The module was evaluated to ensure it supported short-term knowledge gain (research objective 2) and to determine whether module use improved students’ belief in their ability to meet the situational demands of the future pathology laboratory (research objective 1).

#### Study design

The study design was quasi-experimental. The research needed to take place in the course for which the module was designed to best reflect its real-life use and to increase participation. In this setting, it was not allowable or ethical to conduct a randomized controlled study. Therefore, all first-year medical students were allowed in the study, and students self-selected to participate in each phase of the study. Participants who chose to complete the module and the Respiratory Pathology Self-Efficacy Survey were established as the experimental group. Participants who did not complete the module but did complete the Respiratory Pathology Self-Efficacy Survey were established as the control group, as detailed in the section *Establishment of module non-users as a control group*.

#### Data collection instruments

Data were collected in two phases, as demonstrated in Figure 2. Pre-module and post-module quizzes were embedded in the module and were completed immediately before and after module use, respectively. Collection of the quiz scores and module data began upon opening of the module three days before the Respiratory Pathology Laboratory Session and continued until the module closed on the day of the Respiratory Pathology Laboratory Session (Phase 1). Self-efficacy data were collected through a survey prior to the start of the Respiratory Pathology Laboratory Session (Phase 2). The module was closed prior to the laboratory session to prevent individuals from completing the module after the self-efficacy survey, potentially cross-contaminating results. Participants created their own unique identifier number, known only to the participant, that was used by the researchers to link their responses through each phase.

Study data were collected at two time points (phases). The data collected in each phase is listed in gray in the last line of each box. *Day ‘T’ represents the day during which the pathology laboratory session occurred.

##### Pre- and post-module quizzes

Pre- and post-module quizzes were built into the Qualtrics module. The quizzes contained ten multiple choice items on normal histology covered in the module corresponding to Bloom’s level 1 (knowledge) and 2 (comprehension) from the Blooming Anatomy Tool [33] (adapted for histology). Answers to the pre- and post-module quiz questions, along with the participant’s responses, were automatically generated and displayed after completion of the module and quizzes. Since no instruments were available in the literature to specifically test the module’s content, the questions were developed by a histologist (S.A.). The two quizzes were different from each other, but each student answered all the same questions. Pre- and post-module questions were counterbalanced for content and difficulty. The items were expert validated through piloting and review by twelve expert histologists, who were from outside of OUWB, had a related terminal degree, and had taught respiratory histology in a professional program within the past year.

##### Respiratory pathology self-efficacy survey

Measurement of students’ pathology self-efficacy before the respiratory pathology laboratory session was performed to determine the effect of the online histology module on the students’ perceived abilities to meet the situational demands [30] of the pathology laboratory session.

The Respiratory Pathology Self-Efficacy Survey questions (Appendix 2) were modified slightly for tasks related to histopathology from an original validated survey by Chen et al. [34], which was demonstrated to be theory-based, unidimensional, internally consistent, and stable over time with satisfactory content validity and predictive validity [35]. The internal consistency reliability coefficients reported in Chen et al. ranged 0.85 to 0.90, and the test-retest reliability coefficients reported ranged 0.62 to 0.86 [34]. The participants’ surveys were divided into two groups, module users and non-users, by matching the participant identification numbers.

The participants answered six 5-point Likert-style questions assessing respiratory pathology self-efficacy and two 5-point Likert-style questions assessing general self-efficacy. The maximum achievable pathology self-efficacy score was 30, and the maximum achievable general self-efficacy score was 10. The Self-Efficacy Survey was made available via email.

#### Statistical analyses

Statistical analysis was performed using RStudio (RStudio, Boston, MA). The alpha value for significance was set to 0.05. A paired samples *t*-test and Cohen’s *d* effect size comparison of paired samples were used to analyze module quiz data. Kruskal-Wallis rank sum tests, Spearman rank correlation tests, and Mann-Whitney U tests were used to analyze self-efficacy data because self-efficacy was measured with an ordinal 5-point Likert-like scale. Internal consistency reliability for the self-efficacy survey used in the current study was assessed with Cronbach’s alpha. The relationship between preparedness rank and module use was assessed with a chi-square test for independence.

#### Establishment of module non-users as a control group

**Figure 3.**
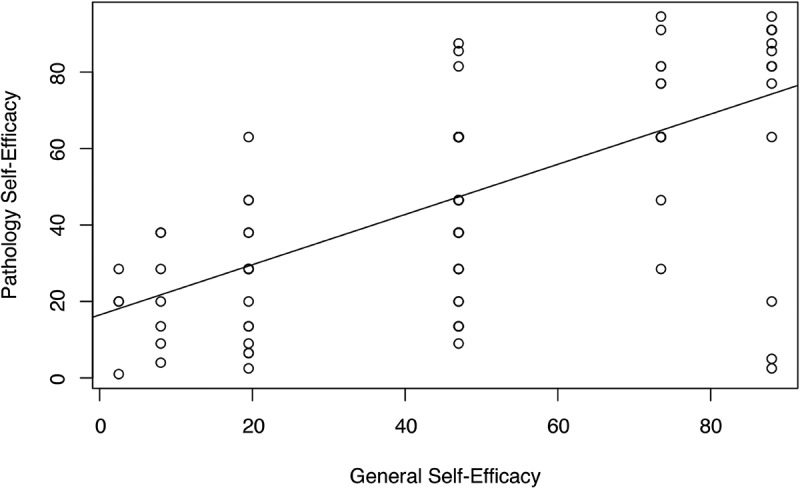
Pathology Self-Efficacy versus General Self-Efficacy

There was a statistically significant positive correlation between pathology self-efficacy and general self-efficacy among all participants (Figure 3), indicating the importance of comparing general self-efficacy between module users and non-users to establish non-users as a control group. There was no statistically significant difference in general self-efficacy between module users and non-users (Table 1A), establishing non-users as an adequate control group. Therefore, the students that did not use the module but chose to participate in the self-efficacy survey were established as the control group.

**Table 1.** Pre-laboratory self-efficacy scores

	Pathology Self-Efficacy			General Self-Efficacy		
*A. Self-Efficacy Scores by Module Use*						
Module Use (*n* = 95)	Median	IQR^§^	MW^†^	Median	IQR^§^	MW^†^
Users (*n* = 62)	24	21–24	*p* = .02*	8	7–9	*p* = .70
Non-users (*n* = 33)	22	19–24		8	7–8	
*B. Self-Efficacy Scores by Preparedness Rank*						
	Pathology Self-Efficacy			General Self-Efficacy		
Preparedness Group (*n* = 95)	Median	IQR^§^	KW^‡^	Median	IQR^§^	KW^‡^
Least Prepared (*n* = 20)	24	22–24.25	*p* = .46	8	7.75–9.25	*p* = .42
Moderately Prepared (*n* = 49)	23	21–24		8	8–9	
Most Prepared (*n* = 26)	22	20–24.75		8	7–9	
*C. Self-Efficacy Scores by Lectures Watched*						
	Pathology Self-Efficacy			General Self-Efficacy		
Lectures Watched (*n* = 95)	Median	IQR^§^	KW^‡^	Median	IQR^§^	KW^‡^
0–1 Lectures (*n* = 13)	24	23–25	*p* = .76	8	8–10	*p* = .27
2–3 Lectures (*n* = 18)	23	21–24		8	7–8	
4–5 Lectures (*n* = 26)	23	20.25–24		8	8–9	
6–7 Lectures (*n* = 38)	22.5	20.25–24		8	7–9	
*D. Self-Efficacy Scores by Self-Study Hours*						
	Pathology Self-Efficacy			General Self-Efficacy		
Self-Study Hours (*n* = 95)	Median	IQR^§^	KW^‡^	Median	IQR^§^	KW^‡^
0 Hours (*n* = 21)	23	21–24	*p* = .59	8	8–9	*p* = .08
1–5 Hours (*n* = 56)	24	21–24		8	7–9	
6–10 Hours (*n* = 16)	22.5	20.75–25.25		8	7–9	
11–15 Hours (*n* = 2)	20.5	20.25–20.75		5	5–5	
>15 Hours (*n* = 0)	–	–		–	–	

^§^Interquartile range; ^†^Mann-Whitney U test; ^‡^Kruskal-Wallis rank sum test; *Significant difference for alpha value set to .05

There is a significant positive correlation between pathology self-efficacy and general self-efficacy among participants (*r*(93) = 0.64, *p* < 0.001, Spearman rank correlation test).

To ensure that any confounding variables were also accounted for, the participants answered four multiple choice questions, as part of the Self-Efficacy Survey, assessing how the student had studied histology and pathology for the course in terms of lectures watched and number of self-study hours in preparation for the laboratory session. Answers to these questions were used to assign students ranks for preparedness outside of module usage. One point was assigned for each of the 7 lectures watched. Zero points were assigned for 0 self-study hours; 1 point was assigned for 1–5 self-study hours; 2 points were assigned for 6–10 self-study hours; 3 points were assigned for 11–15 self-study hours; 4 points were assigned for >15 self-study hours. The participants’ lecture and self-study scores were added together to create the preparedness rank. Participants in the top third of preparedness rank (8–11 total points for preparedness) were considered the ‘most prepared’ group; participants in the second third of preparedness rank (4–7 total points for preparedness) were considered the ‘moderately prepared’ group; participants in the bottom third of preparedness rank (0–3 total points for preparedness) were considered the ‘least prepared’ group. There were no statistically significant differences in pathology or general self-efficacy scores when participants were grouped by preparedness rank (Table 1B), number of lectures watched (Table 1C), and number of self-study hours (Table 1D). There was no statistically significant relationship between preparedness rank and module use (*χ*^2^(10, *n* = 95) = 17.02, *p* = 0.07), indicating that module users and non-users did not differ in their preparation for the laboratory session other than module use.

## Results

Descriptive statistics for module use data and pre- and post- module quiz scores are presented using mean ± standard deviation (SD).

### Module use data

Of the 119 students recruited for the study, there were 79 total module submissions (66% participation). There were 3 repeat module users; however, only 1 of these submissions was marked as ‘finished’ by the software. While the module was open for 76 hours before the pathology laboratory session, the majority of participants completed it in the 5 hours leading up to the laboratory session, with the largest peak at 1 hour before the session. The average time the module was open for each submission marked as ‘finished’ was 1492 ± 648 seconds (~25 ± 11 minutes). Outliers were removed from the dataset to account for students who may have idly left the module open for an extended period of time before completing it.

### Pre- and post-module quizzes

Of 79 total module submissions (Phase 1), 19 submissions were removed due to incompleteness, and 1 from a repeat user was removed. Fifty-nine submissions (75% of all submissions; 50% of all first-year medical students invited) were used for data analysis. Post-module quiz scores (6.8 ± 1.9) were significantly higher (*t*(58) = 3.70, *p* < 0.001) than pre-module quiz scores (5.9 ± 1.7) with a small effect size (*d* = 0.48). This supported the hypothesis that post-histology module quiz scores would be significantly higher than pre-module scores.

### Self-efficacy

In the survey used for this study, the internal consistency reliability coefficients for pathology self-efficacy and general self-efficacy were α = 0.89 and 0.70, respectively, suggesting acceptable internal consistency [36]. Of the 119 students recruited for the study, there were 106 self-efficacy survey submissions during Phase 2 (89% participation). Eleven submissions were removed due to incompleteness. Ninety-five submissions (90% of all submissions; 80% of all first-year medical students invited) were used for data analysis. Of the 95 submissions analyzed, 62 were submitted by individuals who used all or some of the module, and there were 33 non-users. Module users’ pre-laboratory pathology self-efficacy was significantly higher than non-users (*p *= 0.02; Table 1A).

## Discussion

Both research hypotheses were supported by the results: (1) The self-efficacy data indicated that module usage was related to higher self-efficacy in respiratory pathology before the laboratory session; and a similar association was not found between the module users and non-users in their general self-efficacy or preparedness scores. (2) The pre- and post-module quiz scores indicated that module use facilitated short-term knowledge gain, demonstrating that students who used the module had learned from the module.

This study was the first of its kind to determine how an integrative module, created in-house using widely-familiar survey software, influenced medical students’ self-efficacy in one of the integrated disciplines. Although previous studies have explored designing similar histology modules for medical education [13,19,28], they did not explore the use of survey software in module designs, did not focus on integration, and chose different methods to measure the impact of the intervention. For example, Khalil et al. [19] designed a histology module with an e-learning-specific software. Khalil et al. [19], Lei et al. [28], and Thompson and Lowrie [13] measured the impact of the intervention with surveys of students’ perceptions of the module and performance outcomes. The literature has demonstrated that students tend to show positive attitudes and acceptance of self-guided learning modules and technology-enhanced learning [2,19,29,37]. Students’ acceptance of learning materials has continued to prove important in education as students would be less likely to use tools that they perceive as unenjoyable. However, students’ acceptance of resources should not be confused with self-efficacy gained from using resources. Self-efficacy has shown to be an important facet to measure in students because individuals engage in activities and persevere through obstacles when they are confident they will succeed [38].

### Self-efficacy in medical education

Lisbona et al. [39] demonstrated, across multiple organizations, that work engagement and self-efficacy led to higher personal initiative and higher performance. While students’ attitudes towards resources have continued to influence the likelihood of using similar resources in the future, the conclusions from Lisbona et al. [39] could support that self-efficacy gained from such resources would influence how likely students would be to participate in activities learned from that resource in the future. In medical education, students’ willingness to confidently participate in activities could dramatically affect the amount that they learned from the activities. Burgoon et al. [40] postulated a similar theory in regards to self-efficacy in anatomy courses, stating that students with higher anatomical self-efficacy may choose to take a more active role in anatomy dissections. Students that carry out dissections often master the experiences more than students that do not [40]. Therefore, given that participants in the current study had statistically significant higher pathology self-efficacy after module usage, it could be hypothesized that module users would be more likely to actively participate in the subsequent pathology laboratory session and gain more from the session than non-users. Increased participation could have included answering more questions, asking more thoughtful questions, leading case discussions with their peers, and developing extensive differential diagnoses.

Further highlighting the effect of self-efficacy in educational engagement, Demirören et al. [41] demonstrated a positive correlation between self-regulated learning and self-efficacy in problem-based learning. Self-regulated learning is an essential skill for physicians to take responsibility for their own learning after graduating from training and to stay up-to-date in patient care [41]. Problem-based learning helps students integrate different subject matters and develop effective problem-solving skills [42], forming the foundation for clinical reasoning. Therefore, interventions that increase self-efficacy and improve learning skills can enhance students’ abilities in problem-solving and self-regulated learning. In addition, recent studies by Lee and Jeon [43] and Yu et al. [44] demonstrated that academic self-efficacy was negatively correlated with academic burnout. Therefore, it could be postulated that interventions leading to improved self-efficacy may combat student burnout.

### Strengths of the study

The evaluation of this project had several strengths. Because the study took place during an active course and aligned with curricular content, students participated because they saw value in completing the module. Students’ use of the module more closely reflected their real behaviors in an active course compared to a randomized controlled experiment in a supervised setting. The amount of data collected in this study and the sample size allowed for examination of multiple aspects of the relationship between module usage, self-efficacy, and study habits.

### Limitations of the study

A limiting factor in planning this study was that historically there has been low participation in studies conducted outside of the courses to which they pertain. To achieve adequate participation, this study was conducted during an active course, so students could not be randomized into control and experimental groups because it would be unethical to make an academic resource available to select students for an active course. Therefore, a quasi-experimental study design was used instead of the preferred cross-over study design. As a consequence, the size of groups was not controlled for each phase of the study and not exact, but they were similar enough to draw adequate comparisons. The inability to randomize control and experimental groups made the preliminary results susceptible to selection bias.

This study lacked sufficient data to conduct an analysis on performance outcomes. The improvement in post-module quiz scores shows that students retained some knowledge short-term, but this outcome is expected for any pre/post design and does not reveal any new information in long-term knowledge retention. This assessment also cannot compare module users to non-users. An optional post-laboratory pathology quiz was distributed to students but had little participation. Therefore, it was omitted from the analysis for this paper.

### Future directions

Future directions should focus on using the current module as an example to improve integration while increasing student engagement with online active learning activities, especially during a time that students lack integrated ‘hands-on’ learning experiences due to the COVID-19 pandemic [45]. A similar module design would likely be effective in integrating other disciplines in medicine, such as integration of anatomy with medical imaging or embryology with developmental pathologies. Several open-source image-based resources exist for the other anatomical sciences and could be explored for use in modules focusing on other disciplines. Such resources have been curated on the Free Online Anatomy Resources webpage [46] and the American Association for Anatomy’s Anatomy Education Resources webpage [47]. In addition to the *multiple choice; matrix table*; and *pick, group, and rank* question types used in this module, Qualtrics XM also has *hot spot, heat map*, and other question types that would be useful in designing interactive anatomy tools. Further evaluation of the effects of similar modules on performance outcomes and self-efficacy using a cross-over study design could establish the reliability of these learning methods in improving integration without the concern for selection bias that occurred in this study.

Future studies should be conducted to explore the relationship between self-efficacy and post-intervention assessment performance. While there are many studies that have investigated self-efficacy and performance [35,40,48–52], there have been mixed results on the relationship. A study could also be conducted to track changes in self-efficacy before and after interventions that integrate topics. Another future direction could include the addition of a survey later in the respiratory course to compare how module users versus non-users felt about the amount of time they had to study and their overall perceptions on the study materials available to them in the course. An attempt could then be made to draw conclusions on the relationship between module use and efficiency of knowledge attainment in pre-clinical and clinical years.

## Conclusion

Interactive, online modules can be developed affordably and with ease using Qualtrics XM to integrate the anatomical sciences with other disciplines. The current module was developed to bridge the gap between histology and pathology in a respiratory system course by focusing the module on normal versus abnormal morphology. Module use facilitated short-term knowledge gain. Module use facilitated improved pathology self-efficacy before the respiratory pathology laboratory session, while the participants’ general self-efficacy and studying outside of the module did not demonstrate significant influence. Future directions should focus on using the current module as an example to improve integration while increasing student engagement with hands-on active learning activities.

## Data Availability

Data from this manuscript were presented in part in a poster presentation at the AAMC Central Group on Educational Affairs Spring Meeting in Grand Rapids, MI in March 2019.

## Appendix A. Outline of Module Video Content

**Table:**

Video	Normal Histology Featured	Pathology Correlation
1. Respiratory Epithelium	Respiratory epithelium is classified as pseudostratified ciliated columnar from the nasal cavity to distal bronchi (exceptions: nasal vestibules, olfactory region, lingual side of epiglottis, true vocal fold)	Squamous metaplasia is a feature of chronic bronchitis Squamous metaplasia can lead to squamous dysplasia and squamous cell carcinoma
	Tall ciliated cells & function	Smoking interferes with ciliary action, preventing mucus clearance
	Scattered goblet cells & function	Goblet cell hyperplasia is a feature of chronic bronchitis and asthma
	Short neuroendocrine/Kulchitsky cells & function	Origin of neuroendocrine tumors e.g., small cell carcinoma, carcinoid
2. Bronchus	Lumen clear of neutrophils	Neutrophils in lumen seen in bronchopneumonia
	Visible basement membrane	Thickened basement membrane seen in asthma
	Mucosa can have sparse lymphoid tissue (BALT^1^) composed of lymphocytes and histiocytes.	Mucosal inflammation seen in bronchitis: Neutrophils = acute inflammation Plasma cells & lymphocytes = chronic inflammation
	Thin layer of crisscrossing smooth muscle bundles in mucosa	Smooth muscle layer thickening seen in asthma
	Submucosal glands occupy ~40% of the distance between epithelium and cartilage	>40% in chronic bronchitis, sometimes asthma
3. Bronchiole	Largest bronchioles: Ciliated simple columnar epithelium with scattered goblet cells Smallest bronchioles: ciliated simple cuboidal. Scattered club (Clara) cells & function.	
	No inflammatory cells in mucosa	Mucosal inflammation seen in bronchiolitis
	Thin layer of mucus on epithelial cells but mucus should not clog lumen	Mucus can clog lumen in asthma patients
	Thin layer of crisscrossing smooth muscle bundles	Smooth muscle layer thickening is feature of asthma
4. Alveolus	Approximate diameter is 200um	Alveolar space enlargement seen in emphysema
	The only normal structure within the lumen is the alveolar macrophage. Alveolar macrophage function	Inflammatory exudate within the lumen seen in bacterial pneumonia and ARDS^2^ Cellular debris in lumen seen in ARDS Exudate of neutrophils, erythrocytes, and fibrin in lumen seen in bacterial lobar pneumonia in hepatization stage. Clear, acellular fluid in lumen seen in pulmonary edema
	Type I and II pneumocytes & function. Alveolar wall lined by type I and II pneumocytes only.	Neoplastic cells in alveolar wall can lead to adenocarcinoma Pink bands (hyaline membranes) lining the inside of the alveolus can be seen in ARDS and in severe viral pneumonia.
	Thin alveolar septum comprised of type I pneumocytes and their basement membrane sandwiching: Continuous capillaries and their basement membrane Interstitium (rich in elastic, and reticular fibers)	Thickened interalveolar septum seen in viral pneumonia (interstitial pneumonia). Increased mononuclear cells (lymphocytes, plasma cells) present in thickened alveolar walls in viral infection. Fibrosis or increased inflammation in the interstitium can lead to interstitial lung disease

^a^BALT: Bronchus-associated lymphoid tissue; ^2^ARDS: Acute respiratory distress syndrome

## Appendix B. Respiratory Pathology Self-Efficacy Survey^1^

**Table:**

Statement	Strongly disagree	Somewhat disagree	Neither agree nor disagree	Somewhat agree	Strongly agree
I will be able to achieve most of the goals in respiratory histopathology that have been set for me.	□	□	□	□	□
When facing difficult tasks in respiratory histopathology, I am certain that I will accomplish them.	□	□	□	□	□
In general, I think that I can obtain outcomes that are important for histopathology.	□	□	□	□	□
I believe I can succeed at most any endeavor to which I set my mind.	□	□	□	□	□
I will be able to successfully overcome many challenges in respiratory histopathology.	□	□	□	□	□
I am confident that I can perform effectively on many different tasks in respiratory histopathology.	□	□	□	□	□
Compared to other people, I can do most tasks in histopathology very well.	□	□	□	□	□
Even when things are tough, I can perform quite well.	□	□	□	□	□

^1^This self-efficacy survey has been modified for tasks related to histopathology from an original validated self-efficacy survey by Gilad Chen, Stanley M. Gully, and Dov Eden published in *Organizational Research Methods* (Vol. 4, No. 1) pp. 62–83, copyright © 2001 SAGE Publications, Inc. Reprinted by Permission of SAGE Publications, Inc.
